# Multiscale Simulation of Composite Structures: Damage Assessment, Mechanical Analysis and Prediction

**DOI:** 10.3390/ma15186494

**Published:** 2022-09-19

**Authors:** Stelios K. Georgantzinos

**Affiliations:** Laboratory for Advanced Materials, Structures, and Digitalization, Department of Aerospace Science and Technology, National and Kapodistrian University of Athens, 34400 Psachna, Greece; sgeor@uoa.gr

**Keywords:** composite structures, multiscale simulation, finite element analysis, damage assessment, mechanical analysis

## Abstract

Composites can be engineered to exhibit high strength, high stiffness, and high toughness. Composite structures have been used increasingly in various engineering applications. In recent decades, most fundamentals of science have expanded their reach by many orders of magnitude. Currently, one of the primary goals of science and technology seems to be the quest to develop reliable methods for linking the physical phenomena that occur over multiple length scales, particularly from a nano-/micro-scale to a macroscale. The aim of this Special Issue is to assemble high quality papers that advance the field of multiscale simulation of composite structures, through the application of any modern computational and/or analytical methods alone or in conjunction with experimental techniques, for damage assessment or mechanical analysis and prediction.

To engineer composites for high performance and to design advanced structures, the relationship between material nano-/micro-structures and their macroscopic properties must be established in order to accurately predict their mechanical performance and failure. Multiscale simulation is a tool that enables the study and comprehension of complex systems and phenomena that would otherwise be too expensive or dangerous, or even impossible, to study by direct experimentation and, thus, to deal with.

The mechanical characterization of textile composites is a challenging task due to their nonuniform deformation and complicated failure phenomena. However, Zhao et al. [1] introduced a three-dimensional mesoscale finite element model to investigate the progressive damage behavior of a notched single-layer triaxially braided composite subjected to axial tension. The damage initiation and propagation in fiber bundles were simulated using three-dimensional failure criteria and the damage evolution law. A traction–separation law was applied to predict the interfacial damage of fiber bundles. The proposed model was correlated and validated by the experimentally measured full field strain distributions and effective strength of the notched specimen. The progressive damage behavior of the fiber bundles was studied by examining the damage and stress contours at different loading stages. Parametric numerical studies were conducted to explore the role of modeling parameters and geometric characteristics on the internal damage behavior and global measured properties of the notched specimen. Moreover, the correlations of damage behavior, global stress–strain response, and the efficiency of the notched specimen were discussed in detail. The results of this paper delivered a throughout understanding of the damage behavior of braided composites and can help in the specimen design of textile composites.

Accelerated construction in the form of steel–concrete composite beams is among the most efficient methods to construct highway bridges. One of the main problems with this type of composite structure, which has not yet been fully clarified in the case of continuous beam, is the crack zone initiation that gradually expands through the beam width. Gautam et al. [2] proposed a semi-empirical model to predict the size of cracks in terms of small box girder deflection and intensity of the load applied on a structure. A set of steel–concrete composite small box girders were constructed using steel fibrous concrete and experimentally tested under different caseloads. The results were then used to create a dataset of the box girder response in terms of beam deflection and crack width. The dataset obtained was then utilized to develop a simplified formula providing the maximum width of cracks. The results showed that the cracks were initiated in the hogging moment region when the load exceeded 80 kN. Additionally, it was observed that the maximum cracked zone occurred in the center of the beam due to the maximum negative moment. Moreover, the crack width of the box girder at different loading cases was compared with the test results obtained from the literature. A good agreement has been found between the proposed model and experiment results.

With the aim of damage reduction in person subjected to ballistic impact, polymeric, carbon, and glass fibers are commonly used to develop protective systems. However, during recent decades, it has been found that a combination of high strength yarns in different directions generates flexible woven fabrics, which are light and highly resistant at the same time. Therefore, aramid fibers are one of the protection materials most used today, with a growing trend in the industry. Feito et al. [3] investigated the effect of the impact angle of a projectile during low-velocity impact on Kevlar fabrics using a simplified numerical model. The implementation of mesoscale models is complex and usually involves a long computation time, in contrast with practical industry needs to obtain accurate results rapidly. In addition, when the simulation includes more than one layer of composite ply, the computational time increases even in the case of hybrid models. With the goal of providing useful and rapid prediction tools to the industry, a simplified model has been developed in this work. The model offers an advantage in the reduced computational time compared with a full 3D model (around 90% faster). The proposed model has been validated against equivalent experimental and numerical results reported in the literature, with acceptable deviations and accuracies for the design requirements. The proposed numerical model allows for the study of the influence of the geometry on the impact response of the composite. After a parametric study related to the number of layers and the angle of impact, using a response surface methodology, a mechanistic model and a surface diagram were presented to help with the calculation of the ballistic limit.

Concrete-filled steel tubes (CFSTs) show advantageous applications in the field of construction, especially for a high axial load capacity. The challenge in using such structures lies in the selection of many parameters constituting CFST, which necessitates defining complex relationships between the components and the corresponding properties. The axial capacity (Pu) of CFST is among the most important mechanical properties. Nguyen et al. [4] investigated the possibility of using a feedforward neural network (FNN) to predict Pu. Furthermore, an evolutionary optimization algorithm, namely invasive weed optimization (IWO), was used for tuning and optimizing the FNN weights and biases to construct a hybrid FNN–IWO model and to improve its prediction performance. The results showed that the FNN–IWO algorithm is an excellent predictor of Pu, with a value of R^2^ of up to 0.979. The advantage of FNN–IWO was also pointed out with gains in accuracy of 47.9%, 49.2%, and 6.5% for root mean square error (RMSE), mean absolute error (MAE), and R^2^, respectively, compared with the simulation using the single FNN. Finally, the performance when predicting Pu as a function of structural parameters, such as the depth/width ratio, the thickness of the steel tube, the yield stress of steel, the concrete compressive strength, and the slenderness ratio, was investigated and discussed.

Circular opening steel beams have been increasingly acknowledged in structural engineering because of their many remarkable advantages, including their ability to bridge the span of a large aperture or their lighter weight compared with conventional steel beams. Nguyen et al. [5] investigated and selected the most suitable parameters used in particle swarm optimization (PSO), namely the number of rules (n_rule_), the population size (n_pop_), the initial weight (w_ini_), the personal learning coefficient (c_1_), the global learning coefficient (c_2_), and the velocity limits (f_v_), in order to improve the performance of the adaptive neuro-fuzzy inference system in determining the buckling capacity of circular opening steel beams. This is an important mechanical property in terms of the safety of structures under subjected loads. An available database of 3645 data samples was used for the generation of training (70%) and testing (30%) datasets. Monte Carlo simulations, which are natural variability generators, were used in the training phase of the algorithm. Various statistical measurements, such as root mean square error (RMSE), mean absolute error (MAE), Willmott’s index of agreement (IA), and Pearson’s coefficient of correlation (R), were used to evaluate the performance of the models. The results of the study show that the performance of ANFIS optimized by PSO (ANFIS-PSO) is suitable for determining the buckling capacity of circular opening steel beams but is very sensitive under different PSO investigation and selection parameters. The findings of this study show that n_rule_ = 10, n_pop_ = 50, w_ini_ = 0.1 to 0.4, c_1_ = [1, 1.4], c_2_ = [1.8, 2], and f_v_ = 0.1, which are the most suitable selection values for ensuring the best performance for ANFIS-PSO. In short, this study might help in selecting suitable PSO parameters for the optimization of the ANFIS model.

Commonly, nanocomposite material applications are associated with the simultaneous actions with more than one type of loading. Specifically, the investigation of nanocomposites subjected to both thermal as well as mechanical loads is perhaps one of most interesting fields of research, since high-temperature applications are very frequent. Giannopoulos et al. [6] provided a computationally efficient and reliable hybrid numerical formulation capable of characterizing the thermomechanical behavior of nanocomposites, which was based on a combination of molecular dynamics (MD) and the finite element method (FEM). A polymeric material was selected as the matrix—specifically, poly(methyl methacrylate) (PMMA), commonly known as Plexiglas, due to its extensive applications. On the other hand, the fullerene C_240_ was adopted as a reinforcement because of its high symmetry and suitable size. The numerical approach was performed at two scales. First, an analysis was conducted at the nanoscale level by utilizing an appropriate nanocomposite unit cell containing C_240_ at a high mass fraction. A MD-only method was applied to accurately capture all of the internal interfacial effects and, accordingly, its thermoelastic response. Then, a micromechanical, temperature-dependent finite element analysis took place using a representative volume element (RVE), which incorporated the first-stage MD output, to study nanocomposites with small mass fractions, for which a atomistic-only simulation would require a substantial computational effort. To demonstrate the effectiveness of the proposed scheme, numerous numerical results were presented, while the investigation was performed in a temperature range that included the PMMA glass transition temperature, Tg.

Although some work has already been conducted on the vibrations of composite structures reinforced by nanoparticles, there are only a few studies that focused on the vibration behavior of carbon fiber-based laminate composites with pure graphene inclusions. Georgantzinos et al. [7] developed a computational procedure to investigate the vibration behavior of laminated composite structures, including graphene inclusions in a matrix. Concerning the size-dependent behavior of graphene, its mechanical properties were derived using nanoscopic empiric equations. Using the appropriate Halpin–Tsai models, the equivalent elastic constants of the graphene reinforced matrix were obtained. Then, the orthotropic mechanical properties of a composite lamina of carbon fibers and hybrid matrix can be evaluated. Considering a specific stacking sequence and various geometric configurations, carbon fiber-graphene-reinforced hybrid composite plates were modeled using conventional finite element techniques. Applying simply support or clamped boundary conditions, the vibrational behavior of the composite structures was finally extracted. Specifically, the modes of vibration for every configuration were derived, and the effect of graphene inclusions in the natural frequencies was calculated. The higher the volume fraction of graphene in the matrix, the higher the natural frequency for every mode. Comparisons with other methods, where possible, were performed to validate the proposed method.

Giannopoulos and Georgantzinos [8] investigated the thermomechanical effects of adding a newly proposed nanoparticle within a polymer matrix such as polyethylene. A nanoparticle was formed by a typical single-walled carbon nanotube (SWCNT) and two equivalent giant carbon fullerenes that were attached by their nanotube edges through covalent bonds. In this way, a bone-shaped nanofiber that may offer enhanced thermomechanical characteristics when used as a polymer filler, due to each unique shape and chemical nature, was developed. The investigation was based on molecular dynamics simulations of the tensile stress–strain response of the polymer nanocomposites under a variety of temperatures. The thermomechanical behavior of the bone-shaped nanofiber-reinforced polyethylene was compared with that of an equivalent nanocomposite filled with ordinary capped single-walled carbon nanotubes to reach some coherent fundamental conclusions. That study focused on the evaluation of some basic, temperature-dependent properties of the nanocomposite reinforced with these innovative bone-shaped allotropes of carbon.

The flexural strength of Slender steel tube sections is known to achieve significant improvements upon being filled with concrete; however, this section is more likely to fail by buckling under compression stresses. Al Zand et al. [9] investigated the flexural behavior of a Slender steel tube beam that was produced by connecting two pieces of C-sections and filled with recycled-aggregate concrete materials (CFST beam). The C-section’s lips behaved as internal stiffeners for the CFST beam’s cross section. A static flexural test was conducted on five large-scale specimens, including one specimen that was tested without concrete (hollow specimen). The ABAQUS software was also employed for the simulation and non-linear analysis of 20 additional CFST models in order to further investigate the effects of varied parameters that were not tested experimentally. The numerical model was able to adequately verify the flexural behavior and failure mode of the corresponding tested specimen, with an overestimation of the flexural strength capacity of about 3.1%. Generally, the study confirmed the validity of using the tubular C-sections in the CFST beam concept, and their lips (internal stiffeners) led to significant improvements in the flexural strength, stiffness, and energy absorption index. Moreover, a new analytical method was developed to specifically predict the bending (flexural) strength capacity of the internally stiffened CFST beams with steel stiffeners, which was well-aligned with the results derived from the current investigation and with those obtained by others.

The composite shear wall has various merits over traditional reinforced concrete walls. Thus, several experimental studies have been reported in the literature to study the seismic behavior of composite shear walls. However, few numerical investigations were found in previous literature because of difficulties in the interaction behavior of steel and concrete. Najm et al. [10] presented a numerical analysis of smart composite shear walls that use an infilled steel plate and concrete. The study was carried out using the ANSYS software. The mechanical mechanisms between the web plate and concrete were investigated thoroughly. The results obtained from the finite element (FE) analysis show excellent agreement with the experimental test results in terms of the hysteresis curves, failure behavior, ultimate strength, initial stiffness, and ductility. The results indicate that increasing the gap between the steel plate and the concrete wall from 0 mm to 40 mm improved the stiffness by 18% compared with the reference model, which led to delaying failures in this model. Expanding the infill steel plate thickness to 12 mm enhanced the stiffness and energy absorption at ratios of 95% and 58%, respectively. This resulted in a gradual decrease in the strength capacity of this model. Meanwhile, increasing the concrete wall thickness to 150 mm enhanced the ductility and energy absorption at ratios of 52% and 32%, respectively, which led to restricting the model and reducing the lateral offset. Changing the distance between shear studs from 20% to 25% enhanced the ductility and energy absorption by about 66% and 32%, respectively.

A major part of the computational cost required for determining an optimal material design with extreme properties using a topology optimization formulation is devoted to solving the equilibrium system of equations derived through the implementation of the finite element method (FEM). To reduce this computational cost, among other methodologies, various model order reduction (MOR) approaches can be utilized. Kazakis and Lagaros [11] presented a simple Matlab code for solving the topological optimization for the design of materials combined with three different model order reduction approaches. The three MOR approaches presented in the code implemented are the proper orthogonal decomposition (POD), the on-the-fly reduced order model construction and the approximate reanalysis (AR) following the combined approximations approach. The complete code, containing all participating functions (including the changes made to the original ones), was also provided.
